# A Pregnant Woman with Excess Vasopressinase-Induced Diabetes Insipidus Complicated by Central Diabetes Insipidus like Lymphocytic Infundibulo-Neurohypophysitis

**DOI:** 10.1155/2024/8687054

**Published:** 2024-04-13

**Authors:** Shinnosuke Yanagisawa, Yoichi Oikawa, Mai Endo, Kazuyuki Inoue, Ritsuko Nakajima, Shigemitsu Yasuda, Masayasu Sato, Naoko Iwata, Haruki Fujisawa, Atsushi Suzuki, Yoshihisa Sugimura, Masashi Isshiki, Akira Shimada

**Affiliations:** ^1^Department of Endocrinology and Diabetes, Saitama Medical University, 38 Morohongo, Moroyama, Iruma, Saitama 350-0495, Japan; ^2^Department of Obstetrics and Gynecology, Saitama Medical University, 38 Morohongo, Moroyama, Iruma, Saitama 350-0495, Japan; ^3^Department of Endocrinology and Diabetes, Daido Hospital, 9 Hakusui-cho, Minami-ku, Nagoya, Aichi 457-8511, Japan; ^4^Department of Endocrinology, Diabetes and Metabolism, Fujita Health University, 1-98 Dengakugakubo, Kutsukake-cho, Toyoake, Aichi 470-1192, Japan

## Abstract

**Background:**

Gestational diabetes insipidus (DI) is a very rare complication of pregnancy. We present a case of gestational DI combining two different types of DI. *Case Presentation*. A 39-year-old pregnant woman suddenly presented with thirst, polydipsia, and polyuria after 31 gestation weeks (GWs). Based on laboratory findings of hypotonic urine (78 mOsm/kgH_2_O) with higher plasma osmolality (298 mOsm/kgH_2_O) and higher serum sodium levels (149 mEq/L), gestational DI was suspected, and the clinical course was monitored without therapy until the results of a measurement of plasma arginine vasopressin (AVP) levels were available. However, she subsequently developed acute prerenal failure and underwent an emergency cesarean section at 34 GWs. Her resected placenta weighed 920 g, nearly twice the normal weight. Immediately following delivery, intranasal 1-desamino-8-D-arginine vasopressin was administered, and her symptoms promptly disappeared. Afterward, her predelivery plasma AVP level was found to have been inappropriately low (0.7 pg/mL) given her serum sodium level. The patient's serum vasopressinase level just before delivery was 2,855 ng/mL, more than 1,000 times the upper limit of the normal range, suggesting excess vasopressinase-induced DI. The presence of anti-rabphilin-3A antibodies in the patient's blood, a hypertonic saline infusion test result, and loss of the high-intensity signal of the posterior pituitary on fat-suppressed T1-weighted magnetic resonance images without thickening of the stalk and enlargement of the neurohypophysis suggested concurrent central DI-like lymphocytic infundibulo-neurohypophysitis (LINH).

**Conclusion:**

In addition to the degradation of AVP by excess placental vasopressinase due to the enlarged placenta, an insufficient compensatory increase in AVP secretion from the posterior pituitary gland due to LINH-like pathogenesis might have led to DI symptoms.

## 1. Introduction

Diabetes insipidus (DI) during pregnancy, or gestational DI, is a very rare complication of pregnancy, with an estimated incidence of up to 1 in 30,000 pregnancies in the West [1] and 0.81 in 100,000 pregnancies in Japan [2]. Gestational DI is mainly divided into the following three categories: central (neurogenic) DI, nephrogenic DI, and excess vasopressinase-induced DI [2]. In general, central DI is characterized by polyuria and polydipsia due to a deficiency in arginine vasopressin (AVP) and is associated with various underlying diseases including tumors, infiltrative diseases, vascular lesions, and genetic defects that affect the hypothalamic-neurohypophysial system [3, 4]. However, up to 15% of the central DI is reportedly idiopathic [4], and lymphocytic infundibulo-neurohypophysitis (LINH) is considered a common cause of this idiopathic DI [3]. Meanwhile, although vasopressinase, which can degrade AVP, is known to be produced by normal placentae, excess vasopressinase activity may occasionally lead to transient DI during pregnancy due to enhanced degradation of AVP [5]. Here, we present the case of a pregnant woman who complained of thirst, polydipsia, and polyuria due to excess vasopressinase-induced DI concomitantly with central DI, possibly associated with the pathogenesis similar to LINH, followed by acute prerenal failure due to dehydration and the need for an emergency cesarean section.

## 2. Materials and Methods

### 2.1. Hypertonic Saline Infusion Test

A hypertonic saline infusion test was performed after an overnight fast with free water intake. The patient rested in a supine position from 30 min before the beginning of infusion to the end of the test. An indwelling catheter was inserted in the forearm vein at 8:00 am, followed by an intravenous infusion of 5% saline at a rate of 0.05 mL/kg/min for 120 min. Blood samples were collected before and 60 and 120 min after the beginning of the infusion, and plasma AVP and serum sodium levels were measured. Plasma AVP levels were measured by using a radioimmunoassay (RIA) kit (Yamasa Shoyu Corporation, Choshi, Japan), and the measurement was outsourced to SRL Inc. (Tokyo, Japan), a clinical laboratory.

According to a previous study by using the Yamasa RIA kit [6], the simple linear regression analysis was performed to model the relationship between plasma AVP and serum sodium levels, in order to make a diagnosis of central DI. The equation of this simple linear regression line can be represented as “*Y* = *a* + bX”, where *Y* = dependent variable (plasma AVP level), *a* = *Y*-intercept, *b* = slope of the regression line, and *X* = independent variable (serum sodium level). The equation was determined by using a calculation tool openly available on the relevant website (https://kannoukasuitai.jp/academic/cdi/index.html) [6]. A slope (=*b*) of “<0.1” and an estimated plasma AVP level of “<1.0 pg/mL” at a serum sodium level of 149 mEq/L calculated using the above equation were used as diagnostic criteria for central DI [6].

### 2.2. Measurement of Serum Vasopressinase Level

Serum vasopressinase level, synonymous with “leucyl-cystinyl aminopeptidase,” was measured using a Human Leucyl-Cystinyl Aminopeptidase Enzyme-Linked Immunosorbent Assay Kit (MyBioSource, San Diego, CA, USA), according to the manufacturer's instructions.

### 2.3. Detection of Serum Anti-Rabphilin-3A Antibodies

Anti-rabphilin-3A antibodies in the serum were detected by western blotting, as previously described [4, 7]. In brief, a vector containing the full-length human rabphilin-3A gene was transfected into HEK293FT cells to produce a recombinant human rabphilin-3A protein. As a negative control, the same vector without the rabphilin-3A gene was transfected into HEK293FT cells. Anti-rabphilin-3A antibodies in the serum were detected by western blotting using the recombinant human rabphilin-3A protein lysate as the antigen and the serum as the primary antibody. A protein band presenting a size of 76 kDa appeared in the lysate of cells transfected with rabphilin-3A protein but not in that of control cells, which was considered positive for anti-rabphilin-3A antibodies.

## 3. Case Presentation

Two years prior to this report, a 37-year-old female, who was naturally pregnant with her first child, developed polydipsia and polyuria of about 3 L/day late into her pregnancy. Although her pregnancy thereafter proceeded without therapeutic intervention for her symptoms, complications suggesting pregnancy-related hypertensive disorders emerged. As a result, she was delivered by cesarean section at 40 gestational weeks (GWs). Soon after the delivery, her symptoms spontaneously disappeared.

**Figure 1:**
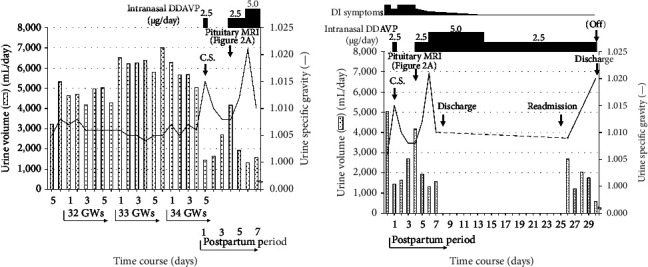
Clinical course of the patient. (a) Clinical course during the perinatal period. Around the end of 31 weeks of gestation, the patient suddenly presented with thirst, polydipsia, and polyuria and was referred to our endocrinology section at 34 weeks and one day of gestation. Although gestational diabetes insipidus was initially suspected, we closely monitored her clinical course without therapy. At 34 weeks and five days of gestation, because of the possible development of acute prerenal failure, an emergency cesarean section (CS) was performed, immediately followed by an initiation of intranasal administration of 1-desamino-8-D-arginine vasopressin (DDAVP). The patient's daily urine volume promptly decreased, along with an increase in specific gravity. (b) Clinical course after delivery. This patient was once discharged on the 8th day postpartum and readmitted to our hospital for pituitary function evaluation, including a hypertonic saline infusion test, on the 25th day postpartum. Thus, no data for daily urine volume and urine-specific gravity from the 8–25th day postpartum were available (dotted line). Although the intranasal administration of DDAVP was discontinued on the 30th day postpartum, the patient remained DI-related symptom-free thereafter. The dotted bar and solid line represent daily urine volume and urine-specific gravity, respectively. CS: cesarean section; DDAVP: 1-desamino-8-d-arginine vasopressin; DI: diabetes insipidus; GWs: gestational weeks; MRI: magnetic resonance imaging (hypothalamic-pituitary region).

At the age of 39, i.e., two years after the first delivery, she was pregnant with her second child by *in vitro* fertilization (IVF). At 22 GWs, she was admitted to our hospital due to premature labor and was started on intravenous magnesium sulfate (16.8 g/day) and ritodrine (200 mg/day). Around the end of 31 GWs, she suddenly developed thirst, polydipsia of about 5 L/day, and polyuria of about 5-6 L/day during hospitalization, for which she was referred to our endocrinology section at 34 weeks and one day of gestation (Figure 1(a)).

Her height, weight, and body mass index were 150 cm, 58 kg, and 25.7 kg/m^2^, respectively. Her laboratory examination at 34 weeks and one day of gestation is shown in Table 1. Based on laboratory findings of hypotonic urine (78 mOsm/kgH_2_O) with a high plasma osmolality (298 mOsm/kgH_2_O) and high serum sodium level (149 mEq/L), gestational DI was initially suspected, and we closely monitored her clinical course without therapy until the results of a measurement of her plasma AVP levels were available. At this point, she suffered from mild liver dysfunction, probably caused by the administration of magnesium sulfate and/or ritodrine (Table 1).

At 34 weeks and five days of gestation, based on an increase in serum creatinine levels from 1.06 mg/dL to 1.45 mg/dL for four days (Table 1), we considered that she might develop progressive renal failure and that it might be dangerous to continue the pregnancy. Thus, an emergency same-day cesarean section was performed. The male baby was large for gestational age with a birth weight of 2,704 g, an Apgar score of 4/4/7 (1/5/10 minutes, respectively), and an umbilical arterial blood pH of 7.38. The patient's resected placenta was greatly enlarged and weighed 920 g, which is nearly twice the normal placenta mass of about 500 g at the same GWs for the Japanese population [8].

**Figure 2:**
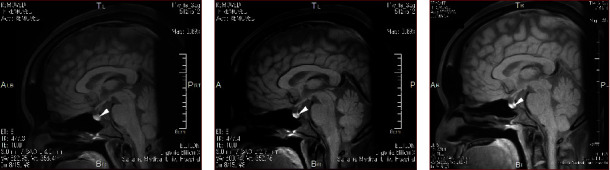
Fat-suppressed T1-weighted magnetic resonance imaging of the hypothalamic-pituitary area. (a) A magnetic resonance imaging (MRI) scan on the fourth day after the delivery showed no obvious abnormal findings in the hypothalamic-pituitary area, except for a loss of the normal bright spot in the posterior lobe (arrowhead). (b) An MRI on the 28th day after the delivery showed a partial reappearance of this bright spot (arrowhead). (c) An MRI on the 201st day after the delivery showed a clear bright spot in the posterior lobe (arrowhead).

Based on the suspicion of acute prerenal failure attributed to DI-related dehydration, intranasal administration of 1-desamino-8-D-arginine vasopressin (DDAVP, a starting dose of 2.5 *µ*g/day) was initiated immediately after the delivery, and the patient's daily urine volume promptly decreased, along with an increase in its specific gravity (Figures 1(a) and 1(b)). Three days after the delivery, pituitary magnetic resonance imaging (MRI) showed a loss of high-intensity signal of the posterior lobe on T1-weighted images with fat suppression, without thickening of the stalk and enlargement of the neurohypophysis (Figure 2(a)). The results of the patient's plasma AVP level measured four days before the delivery (i.e., at 34 weeks and one day of gestation) were then released and found to be inappropriately low (0.7 pg/mL) compared to her high serum sodium level and plasma osmolality (Table 1).

After initiating intranasal administration of DDAVP on the day of delivery, the DI-related symptoms were quickly alleviated and eventually disappeared by the 14^th^ day postpartum (Figure 1(b)). Therefore, the DDAVP dose was reduced from 5.0 *µ*g/day to 2.5 *µ*g/day on the 15^th^ day postpartum. However, no symptom recurrence was observed thereafter (Figure 1(b)). Moreover, although the intranasal administration of DDAVP was discontinued on the 30^th^ day postpartum, the patient remained symptom-free thereafter.

**Figure 3:**
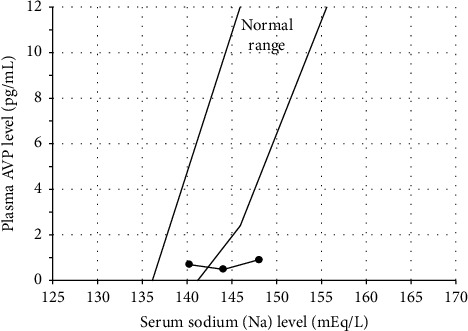
Result of the hypertonic saline infusion test. The relationship between plasma arginine vasopressin (AVP) and serum sodium levels in a 5% hypertonic saline infusion test was performed at four weeks postpartum. Plasma AVP levels did not sufficiently increase in response to the gradual increase in serum sodium levels. Simple linear regression analysis revealed that the equation of the regression line was *Y* = 0.025*X* − 2.9. The slope (=0.025) was <0.1, and the estimated plasma AVP level (=0.825 pg/mL) at a serum sodium level of 149 mEq/mL was <1.0 pg/mL, thus fulfilling the diagnosis criteria for central diabetes insipidus. For reference, the normal range of plasma AVP levels based on the data measured by a former AVP-radioimmunoassay (RIA) kit (Mitsubishi Petrochemical Co. Ltd., Tokyo, Japan), which is now unavailable in Japan, is shown. It is reported that plasma AVP levels measured by the Yamasa RIA kit are significantly and positively correlated with those measured by the Mitsubishi RIA kit (Spearman's rank correlation coefficient (*r*) = 0.905, *p* < 0.001) [9, 10].

**Figure 4:**
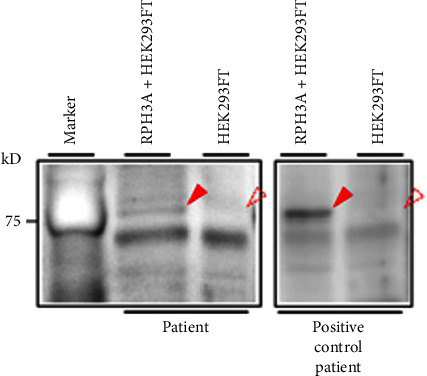
Detection of anti-rabphilin-3A antibodies by western blotting. Recombinant full-length human rabphilin-3A expressed in HEK293FT cells (RPH3A + HEK293FT) or negative control (HEK293FT) were probed with serum from the present patient (patient), or from a patient who was diagnosed with LINH previously (positive control patient). The arrowhead indicates the presence of anti-rabphilin-3A antibodies. The dashed arrowhead indicates the absence of anti-rabphilin-3A antibodies.

At four weeks following delivery, her liver and kidney functions normalized (Table 1). The result of a hypertonic saline infusion test performed at four weeks (on the 27^th^ day) postpartum fulfilled the diagnosis criteria for central DI (Figure 3), and the patient was positive for anti-rabphilin-3A antibodies, a highly sensitive and specific diagnostic marker for LINH (Figure 4) [4, 7]. Meanwhile, fat-suppressed T1-weighted MRI around the same time showed a partial reappearance of a physiological bright spot in the posterior pituitary (Figure 2(b)). In addition, anterior pituitary function tests revealed that the responses of all anterior pituitary hormones were spared (Table 2). An MRI around six months after the delivery showed a clear bright spot in the posterior pituitary, suggesting the recovery of the posterior lobe function (Figure 2(c)). Meanwhile, pituitary MRI findings meeting the criteria for the diagnosis of LINH in Japan, e.g., enlargement of the pituitary gland or stalk on imaging [11], could not be observed during the clinical course.

The patient's serum level of vasopressinase as just one hour before the delivery was 2,855 ng/mL, more than 1,000 times the upper limit of the normal range for the third trimester (0.2–2.0 ng/mL) [12]. Thereafter, her serum vasopressinase level rapidly decreased to 322.9 ng/mL on the sixth day after the delivery. Considering the clinical course described above, the patient was finally diagnosed as having excess vasopressinase-induced DI concomitantly with central DI, possibly associated with the pathogenesis similar to LINH.

The patient tested positive for anti-rabphilin-3A antibodies at approximately three years after the delivery (data not shown).

## 4. Discussion

We encountered a pregnant woman with both excess vasopressinase-induced DI, in which the degradation of AVP by the enlarged placenta-derived excess vasopressinase might have resulted in a decrease in plasma AVP levels, and central DI possibly involving autoimmune mechanism such as LINH. Dehydration and acute prerenal failure resulted in necessitating an emergency cesarean section. To the best of our knowledge, this is the first case of the copresentation of these two types of DI.

In normal pregnancies, vasopressinase, a cysteine aminopeptidase that degrades AVP by sequentially removing amino acids from the N-terminus and therefore increases AVP clearance [13], is produced starting from around seventh GW by placental trophoblasts [5, 14–16]. The activity of vasopressinase correlates with the volume of the placenta [5, 14–16]. Thus, its activity increases 40–50-fold during mid (22^nd^ to 24^th^ GW) and late (36^th^ to 38^th^ GW) pregnancy [14, 17]. Accordingly, AVP clearance reaches a plateau at a four-fold higher rate by the 22^nd^–24^th^ GW and remains at this level until delivery [17, 18]. On the other hand, to sufficiently maintain its antidiuretic effect, a compensatory increase in AVP synthesis and secretion by the posterior pituitary gland is usually observed in normal pregnancy, resulting in no manifestation of symptoms of DI [19]. In the end, this enzyme becomes undetectable 5-6 weeks after delivery [17].

Excess vasopressinase activities less frequently lead to transient gestational DI. The prevalence of excess vasopressinase-induced DI is reportedly estimated to be approximately 2–4 in 100,000 pregnancies [5, 16]. This type of DI usually develops in late pregnancy, with chief complaints being thirst, polydipsia, and polyuria. Based on previous reports, individuals with this endocrine disorder can have up to 300 times higher levels of vasopressinase than normal healthy pregnant women [2]. As with normal pregnancies, the excess vasopressinase activity naturally decreases after delivery and becomes undetectable by 5-6 weeks postpartum [5, 19]. Excess vasopressinase-induced DI is caused by the deficiency of endogenous AVP resulting from its degradation by vasopressinase and is usually intractable to treatment with exogenous AVP but is responsive to DDAVP. This is because DDAVP, a synthetic form of AVP characterized by a deaminated N-terminus, is resistant to degradation by vasopressinase [13]. This characteristic responsiveness to DDAVP administration was true for the present patient as well.

Excess vasopressinase-induced DI is more commonly observed in individuals with enlarged placentae as is typically seen in pregnancies with multiple fetuses, or in cases of impaired hepatic degradation of vasopressinase as associated with hepatitis (viral hepatitis, drug-induced hepatitis, and hepatitis of unknown etiology), acute fatty liver of pregnancy (AFLP), and HELLP syndrome (hemolysis, elevated liver enzymes, and low platelet count) [2]. The patient in this case was pregnant with a single child, which could not explain her enlarged placenta. Concomitant liver dysfunction may contribute to enhanced AVP degradation via a decrease in hepatic degradation of vasopressinase. In addition, pregnancies conceived by assisted reproductive technology (ART), including IVF, are reportedly associated with larger placental weights, independent of the length of gestation at delivery [20]. ART treatment includes exogenous FSH stimulation for the development of multiple follicles, leading to elevated levels of estradiol and progesterone from multiple corpora lutea. Since an endometrium exposed to such high levels of female hormones is less receptive to implantation of an embryo than a normal endometrium, a compensatory growth of the placenta is thought to be required for survival of an implanting embryo in such a suboptimal environment [20, 21]. Although the actual incidence remains to be elucidated, IVF-associated pregnancy might have been associated with transient DI in the present patient via overproduction of vasopressinase by the enlarged placenta.

LINH accounts for a significant portion of cases with autoimmune central DI. It is characterized by lymphocytic inflammation of the posterior pituitary and infundibular stalk [3, 4]. Although there have been no data on the precise incidence of LINH, the incidence of lymphocytic hypophysitis, including LINH, is reportedly 1 in 9 million individuals per year [22], suggesting that LINH is extremely rare. In typical cases of LINH, although the anterior pituitary gland and hypothalamus are functionally and morphologically normal, a high-intensity signal corresponding to the posterior gland on T1-weighted images is absent. In addition, almost half of the subjects show thickening of the pituitary stalk, enlargement of the neurohypophysis, or both [3]. According to clinical guidelines of the Japan Endocrine Society, the criteria used to diagnose LINH require the following two imaging findings: (i) enlargement of the pituitary gland or stalk on imaging and (ii) strong and diffuse enhancement in the neurohypophysis and/or stalk lesion on MRI with gadolinium enhancement [11]. Our patient did not show either of these two MRI findings and thus failed to reach a definitive diagnosis of LINH. However, the disappearance of the high-intensity signal of the posterior pituitary gland was observed. Moreover, anti-rabphilin-3A antibodies were detected in her peripheral blood, suggesting the presence of central DI associated with LINH [4, 7]. Thus, in addition to the degradation of AVP by excess placental vasopressinase, an insufficient compensatory increase in AVP secretion by the pituitary posterior gland might have contributed to a decrease in the patient's serum AVP level, leading to the manifestation of DI symptoms. Meanwhile, the inflammatory process in LINH is reportedly self-limited and regresses spontaneously [3], partially accounting for the spontaneous remission seen in this patient.

To clarify the association between the presence or absence of anti-rabphilin-3A antibodies and the recovery of the high-intensity signal of the posterior pituitary gland, we attempted to detect anti-rabphilin-3A antibodies using the patient's peripheral blood approximately three years after the delivery. The antibodies were detected, suggesting that they might have remained in the circulation for at least three years after the delivery, although the DI-related symptoms and MRI findings were in the recovery process. Based on a basic research using a mouse model, LINH is considered a T cell-mediated autoimmune disease, in which T cells specific for rabphilin-3A may be involved in the disease pathogenesis [23]. In this model, the administration of anti-rabphilin-3A antibodies did not induce LINH, suggesting that the antibodies themselves are not directly involved in the pathogenesis [23]. Therefore, the presence of anti-rabphilin-3A antibodies suggests the involvement of LINH-related pathological conditions and can be useful as a diagnostic marker for this disease. However, we believe that the presence of the antibodies may not necessarily reflect the disease activity of LINH, including the MRI findings of the recovery process in the posterior pituitary gland.

When the patient was pregnant with her first child, she experienced polydipsia and polyuria during late pregnancy, which were transient and spontaneously disappeared after delivery. Although there were no data on the volume of her placenta, anti-rabphilin-3A antibodies, and pituitary MRI findings at that time, judging from her transient DI-like symptoms, she might have suffered from mild gestational DI during her first pregnancy as well.

The patient remained DI-related symptom-free even after intranasal DDAVP discontinuation, and the hypertonic saline infusion test met the diagnostic criteria for central DI. The reason for this discrepancy between clinical symptoms and the test result may be related to the fact that the serum AVP level was within the normal range when the serum sodium level was approximately 140 mEq/L before the start of the hypertonic saline infusion test (Figure 3). Therefore, as long as the serum sodium level was within the normal range and the patient has free access to water, the symptoms of central DI may not occur. Meanwhile, insufficient AVP secretion was suspected under the condition of a serum sodium level of approximately ≥143 mEq/L (Figure 3), suggesting that the posterior pituitary function reserve may be partially impaired in this patient. In other words, this patient presented with a partial central DI [24]. Thus, once this patient is placed in a situation in which the serum sodium level increases, such as dehydration without access to free drinking water, the urine cannot be properly concentrated owing to an insufficient secretion of AVP. Moreover, the serum sodium level, i.e., plasma osmolarity, may rapidly increase, presumably leading to the development of DI symptoms. Overall, in this patient, we believe that the serum sodium level might have remained at approximately 140 mEq/L with free access to water even after the discontinuation of intranasal DDAVP, presumably resulting in no DI symptoms despite the fulfillment of central DI diagnostic criteria.

The patient received magnesium sulfate during hospitalization. According to the drug package insert, this drug may cause DI with an unknown frequency, although the mechanism behind this remains unknown. Thus, the administration of magnesium sulfate might have partially contributed to the pathogenesis of DI in this case.

The present patient underwent pituitary function tests at four weeks postpartum, at which time she had been already asymptomatic although with a small dose (2.5 *µ*g/day) of intranasal DDAVP administration (Figure 1(a)). Thus, her actual pituitary functions in the presence of her DI symptoms before delivery remain unknown, disrupting accurate evaluation of the pathogenesis or severity of her late pregnancy DI, which is a limitation of this case report.

## 5. Conclusion

In conclusion, we encountered a pregnant woman with excess vasopressinase-induced DI, concomitant with central DI, possibly associated with LINH, who presented with DI symptoms in late pregnancy. In addition to plasma AVP and serum vasopressinase levels, measurement of serum anti-rabphilin-3A antibodies and pituitary MRI findings may help diagnose the distinct gestational DI by combining these two very rare different types of DI. Furthermore, ART might be associated with an increased risk of excess vasopressinase-induced DI due to enlarged placentae, which requires further investigation. We believe that this case is etiologically interesting because the pathological condition of DI during pregnancy is presumably formed by the additive involvement of two different mechanisms of DI, which prompted us to report this case. This report provides an answer to the question of which clinical conditions contribute to the development and/or worsening of DI symptoms in pregnant women with excess vasopressinase-induced DI. Conventionally, it has been considered that the main pathogenesis of excess vasopressinase-induced DI is the deficiency of endogenous AVP, resulting from its degradation by excess vasopressinase, even though a compensatory increase in AVP secretion by the posterior pituitary gland functions normally. However, we would like to argue that, in some patients with excess vasopressinase-induced DI, an insufficient compensatory increase in AVP secretion, caused by central DI associated with posterior pituitary gland lesions, may latently and additively contribute to the development of DI symptoms associated with excess vasopressinase. Furthermore, the clinically important point is that, in such cases, DI symptoms caused by central DI may persist even after the disappearance of peripheral vasopressinase postpartum, although this was not the case in the present patient. Regarding this point, measuring anti-rabphilin-3A antibodies during pregnancy in women with excess vasopressinase-induced DI may be helpful not only in determining the coexistence of central DI associated with LINH but also in predicting the persistence of DI symptoms after delivery if the antibodies are positive. In cases of gestational DI, considering the possibility of the coexistence of these two different types of DI, as observed in this case, physicians should measure anti-rabphilin-3A antibodies as needed, and if the antibodies are positive, they should prepare to provide adequate therapy for central DI, which may persist even after delivery.

## Figures and Tables

**Table 1:** Laboratory test results before and after delivery.

	At 34 weeks and one day of gestation	At 34 weeks and five days of gestation (just before C.S.)	At four weeks after delivery
*Complete blood count*			
White blood cell (×10^3^/*µ*L)	6.40	13.73	4.36
Red blood cell (×10^6^/*µ*L)	5.50	4.96	4.38
Hemoglobin (g/dL)	12.3	11.4	10.8
Hematocrit (%)	42.6	38.1	3.56
Platelets (×10^3^/*µ*L)	234	124	413
*Biochemistry*			
AST (IU/L)	65	44	14
ALT (IU/L)	55	24	9
*γ*-GTP (IU/L)	49	38	13
LDH (IU/L)	361	489	161
T-Bil (mg/dL)	0.3	0.4	0.4
BUN (mg/dL)	8.8	15.4	11.9
Cre (mg/dL)	1.06	1.45	0.61
TP (g/dL)	5.8	4.2	5.7
Alb (g/dL)	2.6	1.8	3.3
Na (mEq/L)	149	140	142
Cl (mEq/L)	112	106	107
K (mEq/L)	3.8	4.6	4.1
Glucose (mg/dL)	103	74	87
Hemoglobin A1c (%)	—	6.5	5.0
Plasma osmolality (mOsm/kgH_2_O)	298	—	282
*Urinalysis*			
Specific gravity	1.005	1.016	1.009
Urinary osmolality (mOsm/kgH_2_O)	78	—	376
Urinary protein	(−)	(±)	(−)
Urinary glucose	(−)	(−)	(−)
*Hormones*			
GH (ng/mL)	18.84	—	0.18
IGF-1 (ng/mL) (100–250)	260.6	—	118.5
ACTH (pg/mL)	23.7	—	28.5
Cortisol (*µ*g/dL)	15.8	—	7.82
LH (mIU/mL)	0.2	—	2.5
FSH (mIU/mL)	0.1	—	6.3
PRL (ng/mL)	—	—	153.1 (lactation period)
TSH (*µ*IU/mL)	0.65	—	0.70
Free T3 (pg/mL)	2.43	—	2.78
Free T4 (ng/dL)	0.95	—	0.87
AVP (pg/mL)	0.7	—	1.0
*Others*			
IgG (mg/dL)	—	444	—
IgA (mg/dL)	—	139	—
IgM (mg/dL)	—	153	—
Antinuclear antibody	—	(−)	—
Antimitochondrial antibody	—	(−)	—
IgM-HA antibody	—	(−)	—
HBs antigen	—	(−)	—
IgM-HBc antibody	—	(−)	—
HCV antibody	—	(−)	—
CMV-IgM	—	(−)	—
VCA-IgM	—	(−)	—
EBNA-IgG	—	(−)	—

The range of reference values is indicated in the parentheses. ACTH, adrenocorticotropic hormone; Alb, albumin; ALT, alanine aminotransferase; AST, aspartate transaminase; AVP, arginine vasopressin; BUN, blood urea nitrogen; CMV, cytomegalovirus; Cre, creatinine; CS, cesarean section; EBNA, Epstein–Barr nuclear antigen; FSH, follicle stimulating hormone; *γ*-GTP, *γ*-glutamyl transpeptidase; GH, growth hormone; HA, hepatitis A virus; HB, hepatitis B virus; HCV, hepatitis C virus; IGF-1, insulin-like growth factor-1; Ig, immunoglobulin; LDH, lactate dehydrogenase; LH, luteinizing hormone; PRL, prolactin; T3, triiodothyronine; T4, thyroxine; T-Bil, total bilirubin; TP, total protein; TSH, thyroid stimulating hormone; VCA, virus capsid antigen.

**Table 2:** Anterior pituitary function tests at four weeks postpartum.

*CRH/TRH/LHRH loading test*					

Min.	0	30	60	90	120
LH (mIU/mL)	0.7	22.4	19.6	1.53	13.3
FSH (mIU/mL)	4.4	16.7	19.2	19.1	19.2
PRL (ng/mL)	71.9	235.4	163.1	112.9	90.4
TSH (*µ*IU/mL)	0.7	7.3	6.7	5.3	4.2
ACTH (pg/mL)	19.7	125.6	95.8	53.7	38.7
Cortisol (*µ*g/dL)	7.1	19.1	22.9	17.4	15.2

*GHRP-2 loading test*					

Min.	0	15	30	45	60
GH (ng/mL)	0.69	20.6	17.8	11.0	5.3

ACTH, adrenocorticotropic hormone; CRH, corticotropin-releasing hormone; FSH, follicle stimulating hormone; GH, growth hormone; GHRP-2, growth hormone releasing peptide-2; Min., minutes; LH, luteinizing hormone; LHRH, luteinizing hormone-releasing hormone; PRL, prolactin; TRH, thyrotropin-releasing hormone; TSH, thyroid stimulating hormone.

## Data Availability

Data sharing is not applicable.
